# Draft Genome Sequences of Three Amino Acid-Secreting Lactococcus lactis Strains

**DOI:** 10.1128/MRA.00158-20

**Published:** 2020-04-16

**Authors:** Jhonatan A. Hernandez-Valdes, Anne de Jong, Jan Kok, Oscar P. Kuipers

**Affiliations:** a Department of Molecular Genetics, Groningen Biomolecular Sciences and Biotechnology Institute, University of Groningen, Groningen, The Netherlands; University of Arizona

## Abstract

Three Lactococcus lactis strains with the ability to secrete various amino acids (leucine, isoleucine, methionine, valine, glutamic acid, and histidine) were sequenced in order to identify the mechanisms involved in the secretion. Amino acids contribute to flavor formation; therefore, bacterial strains with this ability are relevant for the food industry.

## ANNOUNCEMENT

Bacteria secrete several compounds during growth, as well as in stationary phase. Some of these compounds are relevant for the food industry, for instance in the large-scale production of amino acids that find application as feed additives, flavor-promoting compounds, or ingredients in pharmaceuticals (1 – 3). Moreover, the relationship between amino acids and flavor formation has been studied extensively in lactic acid bacteria used in dairy fermentations, in order to understand and to improve the organoleptic properties of dairy products (4, 5). In particular, Lactococcus lactis is widely used as a starter culture for the manufacture of buttermilk, quark, and a wide variety of cheeses (6). Its proteolytic system provides the cells with essential amino acids from casein (7). The amino acids, obtained from casein degradation, are either flavor compounds or flavor precursors (8, 9).

In this work, we report three amino acid-secreting L. lactis strains from the laboratory collection of the molecular genetics department at the University of Groningen (Groningen, The Netherlands) (J. A. Hernandez-Valdes, manuscript in preparation). The strains were originally isolated from dairy environments. The L. lactis C17 strain was obtained from the NIZO collection, the L. lactis NCDO176 strain was obtained from the DSMZ collection, and the L. lactis WW4 strain was obtained from the MolGen collection. A single colony of each strain growing on an LM17 agar plate was selected, grown as a standing culture in 5 ml of M17 broth supplemented with 0.5% (wt/vol) lactose (LM17 broth), and incubated overnight at 30°C. Cells from the three cultures were collected by centrifugation at 10,000 rpm for 3 min in a Microfuge 16 centrifuge (Beckman Coulter, Woerden, The Netherlands). Genomic DNA was isolated with a GenElute bacterial genome DNA kit (Sigma-Aldrich, Munich, Germany) according to the manufacturer’s instructions.

The genomes of the lactococcal strains were paired-end sequenced at the Beijing Genomics Institute (Copenhagen, Denmark) on a BGISEQ-500 platform. A total of 5 million paired-end reads (150 bp) were generated. FastQC version 0.11.5 (10) was used to examine the quality of the reads, and low-quality reads were removed with Trimmomatic version 0.38 (11). Subsequently, SPAdes version 3.11.1 (12) was used with default parameters to perform a *de novo* paired-end assembly for each genome, resulting in the draft genome sequences. The coverages of the three sequenced genomes all exceeded 150×. The characteristics of the assemblies and genome features obtained are described in Table 1. Taxonomic assignment of reads was performed with Kraken version 2.0.7 (13). The Rapid Annotations using Subsystems Technology (RAST) server (14) and Prokka (15) were used to annotate the genomes. Further analysis of the genomes, in order to discover the mechanisms underlying amino acid secretion by these bacteria, is under way.

**TABLE 1:** Genome features and accession numbers for the three Lactococcus lactis strains

*Lactococcus lactis* subsp. *lactis* strain	Genome size (bp)	G+C content (%)	No. of coding sequences	No. of contigs	GenBank accession no.	SRA accession no.
C17	2,552,877	35.0	2,717	130	WJUK00000000	SRR10203129
NCDO176	2,445,329	35.1	2,579	120	WJUL00000000	SRR10203130
WW4	2,553,867	34.9	2,716	132	WJUM00000000	SRR10203131

### Data availability.

The genome sequences of the three Lactococcus lactis strains have been deposited in GenBank under the accession numbers listed in Table 1. The raw reads were submitted to the Sequence Read Archive (SRA) under the accession numbers listed in Table 1.
